# Editorial Note: Advances in Enzymology and Enzyme Engineering

**DOI:** 10.5936/csbj.201209001

**Published:** 2012-09-28

**Authors:** Paul Christakopoulos, Evangelos Topakas

**Affiliations:** a Biochemical and Chemical Process Engineering, Division of Sustainable Process Engineering, Department of Civil, Environmental and Natural Resources Engineering, Luleå University of Technology, SE-97187Luleå, Sweden; b BIOtechMASS Unit, Biotechnology Laboratory, School of Chemical Engineering, National Technical University of Athens, 5 IroonPolytechniouStr, Zografou Campus, 15700, Athens, Greece

Enzymes have played an important role in many aspects of life since the dawn of time. In fact they are vitally important to the existence of life itself. Civilizations have used enzymes for thousands of years without understanding what they were or how they work. The modern history of enzymes dates back to 1833 when, in the journal Annales de Chemie et de Physique, the French chemists Anselme Payen and Jean-Franois Persoz described the isolation of an amylase complex from germinating barley and named it diastase. Like malt itself, this product converted gelatinized starch into sugars, primarily maltose. In 1835 the Swedish chemist Jöns Jacob Berzelius demonstrated that starch can be broken down more efficiently using malt extract than sulphuric acid and coined the term catalysis. In 1836, while investigating digestive processes, the German physiologist Theodor Schwann isolated a substance responsible for albuminous digestion in the stomach and named it pepsin, the first enzyme prepared from animal tissue. In 1876, William Kuhne proposed that the name ‘enzyme’ be used as the new term to denote phenomena previously known as ‘unorganised ferments’, that is, ferments isolated from the viable organisms in which they were formed. The word itself means ‘in yeast’ and is derived from the Greek ‘en’ meaning ‘in’, and ‘zyme’ meaning ‘yeast’ or ‘leaven’.

The fact that enzymes are a type of protein was discoveredin 1926 by James Sumner, who identified urease as a proteinafter purification and crystallization. Other important contributors to the development of enzyme chemistry include K. Linderstrom-Lang and M. Ottesen, who were the first to isolate and characterize a subtilisin, a type of alkaline protease produced by bacteria.

The use of enzymes in detergents and their largest industrial application began slowly in the early 1930s, based on Rohm's 1913 patent on the use of pancreatic enzymes in presoak solutions; however microbial proteases were first used in detergents during the sixties. In the same period, the commercial availability of glucoamylase made possible the complete enzymatic conversion of corn starch to glucose. These are two major breakthroughs that had a major impact on the enzyme industry. The first commercialized enzyme expressed in a genetically modified organism was a lipase for detergents called LipolaseR. It was developed by Novo and introduced in 1988 for immediate incorporation into the Japanese detergent Hi-Top made by the Lion Corporation.

In the current third wave of biocatalysis, there have been numerous commercial enzymes with remarkable capabilities available for academia and industry. However, the pool of enzymes catering to industrial demands is still insufficient. Hence, it is still necessary to discover and engineer novel and better enzymes. Approaches to discovering and engineering new and better enzymes of industrial potential are rapidly developing. The achievements in microbiology and molecular biology in the eighties have already led to a broad range of widely applicable enzymes showing an excellent performance. Moreover modifying the properties of enzymes and proteins has become a relatively routine practice in both the academic and the bioindustrial sectors, since the first introduction of the concept in the late 70's to early 80s. With the ever increasing amounts of biological information available for genomics programs the original tools of site-specific modification and three dimensional structural analysis based design have expanded to include regio-targeted mutagenesis, homology based recruitment of amino acid residues and *in vivo* or *in vitro* recombination of genes. The advent of PCR based methodologies for gene cloning and manipulations have greatly increased the ability to design altered proteins, while at the same time, have made the process less time-consuming. Moreover, the ability to introduce thousands, if not millions, of variations into existing proteins, the rapid development of robotic based handling and screening systems and one has the basis for a revolutionary excursion into protein design.

The introduction of a new generation of cheap enzymes, with enhanced activities and resilience, should change the economic balance in favor of enzyme use. It gives us a great pleasure to present this special issue. The issue comprises of 20 review articles covering both basic and applied aspects of enzyme engineering, as well as the use of enzymes in the production of various types of bioproducts. We would like to thank the collaboration of all the authors and the reviewers.

